# OneD: increasing reproducibility of Hi-C samples with abnormal karyotypes

**DOI:** 10.1093/nar/gky064

**Published:** 2018-01-31

**Authors:** Enrique Vidal, François le Dily, Javier Quilez, Ralph Stadhouders, Yasmina Cuartero, Thomas Graf, Marc A Marti-Renom, Miguel Beato, Guillaume J Filion

**Affiliations:** 1 Gene Regulation, Stem Cells and Cancer Program, Centre for Genomic Regulation (CRG), The Barcelona Institute of Science and Technology (BIST), Dr. Aiguader 88, 08003, Barcelona, Spain; 2 Universitat Pompeu Fabra (UPF), Barcelona, Spain; 3 CNAG-CRG, Centre for Genomic Regulation (CRG), Barcelona Institute of Science and Technology (BIST), Baldiri i Reixac 4, 08028 Barcelona, Spain; 4 ICREA, Pg. Lluís Companys 23, 08010 Barcelona, Spain

## Abstract

The three-dimensional conformation of genomes is an essential component of their biological activity. The advent of the Hi-C technology enabled an unprecedented progress in our understanding of genome structures. However, Hi-C is subject to systematic biases that can compromise downstream analyses. Several strategies have been proposed to remove those biases, but the issue of abnormal karyotypes received little attention. Many experiments are performed in cancer cell lines, which typically harbor large-scale copy number variations that create visible defects on the raw Hi-C maps. The consequences of these widespread artifacts on the normalized maps are mostly unexplored. We observed that current normalization methods are not robust to the presence of large-scale copy number variations, potentially obscuring biological differences and enhancing batch effects. To address this issue, we developed an alternative approach designed to take into account chromosomal abnormalities. The method, called *OneD*, increases reproducibility among replicates of Hi-C samples with abnormal karyotype, outperforming previous methods significantly. On normal karyotypes, *OneD* fared equally well as state-of-the-art methods, making it a safe choice for Hi-C normalization. *OneD* is fast and scales well in terms of computing resources for resolutions up to 5 kb.

## INTRODUCTION

One of the crown achievements of modern biology was to realize that genomes have an underlying three-dimensional structure contributing to their activity (1–3). In mammals, this organization plays a key role in guiding enhancer-promoter contacts (4), in V(D)J recombination (5) and in X chromosome inactivation (6). A significant breakthrough towards this insight was the development of the high throughput chromosomal conformation capture technology (Hi-C), assaying chromosomal contacts at a genome-wide scale (7). Nowadays, exploring the spatial organization of chromatin has become a priority in many fields and Hi-C has become part of the standard molecular biology toolbox (8).

Contrary to the precursor technologies 3C, 4C and 5C (9–12), Hi-C interrogates all possible pairwise interactions between restriction fragments. However, this does not guarantee that the method has no bias. On the contrary, local genome features such as the G+C content, the availability of restriction enzyme sites and the mappability of the sequencing reads have been shown to impact the results (13), in addition to general experimental biases such as batch effects. It is thus important to normalize Hi-C data in order to remove biases and artifacts, so that they are not confused with biological signal.

Several methods have been proposed to remove biases in Hi-C experiments (14). The first strategy is to model biases explicitly from a defined set of local genomic features, such as the G+C content. This approach is used in the method of (13) and in HiCNorm by (15). The second strategy is to implicitly correct unknown biases by enforcing some regularity condition on the data. This approach is used in the Iterative Correction and Eigenvector decomposition method (*ICE*) of (16), whereby the total amount of contacts of every bin is imposed to be the same. *ICE* is currently the most popular method, due in part to its speed and simplicity.

Neither of these strategies were designed for cell types with karyotypic aberrations, most common in cancer. Yet, Hi-C is very sensitive to aneuploidy, copy number variations and translocations. Actually, these aberrations have so much influence on the outcome that they can be used as signatures to re-assemble the target genome (17). An additional complication is that karyotypic aberrations are not experimental biases, so it is unclear whether they should be corrected at all or be considered part of the biological signal.

So far, the only attempt to address the issue was the chromosome-adjusted Iterative Correction Bias method (*caICB*) of (18). However, *caICB* applies a uniform chromosome-wide copy number correction, effectively excluding the numerous cases of partial aneuploidy and regional copy number variations.

Here, we propose *OneD*, a method to correct local chromosomal abnormalities in Hi-C experiments. *OneD* explicitly models the contribution of known biases via a generalized additive model. The normalized data is more reproducible between replicates and across different protocols. Importantly, *OneD* is also efficient when cells have a normal karyotype, where it performs as well as the best normalization methods. Finally, the implementation is as fast as *ICE* and it scales up to 5 kb resolution with reasonable computing resources.

## MATERIALS AND METHODS

### Model

The most common representation of Hi-C data is a contact matrix, obtained by slicing the genome in *n* consecutive bins of fixed size (the resolution) and computing the number of contacts between each pair of bins. The values are stored in the cells of the contact matrix (*x*_*ij*_), quantifying the interaction frequency between the two loci at positions *i* and *j*.

Our approach is to model the total number of contacts for each bin, thus reducing the matrix to a one-dimension score (hence the name *OneD*) referred to as the ‘contact profile’. We assume that the total number of contacts per bin *t*_*i*_ can be approximated by a negative binomial distribution. This choice is sensible because the amount of contacts is a discrete variable that often presents a variance higher than the mean (19) (a phenomenon known as overdispersion), and because the negative binomial distribution allows for overdispersion (20). We further assume that the explicit sources of bias have independent contributions to the mean of the distribution for a given bin λ_*i*_.

**Figure 1.**
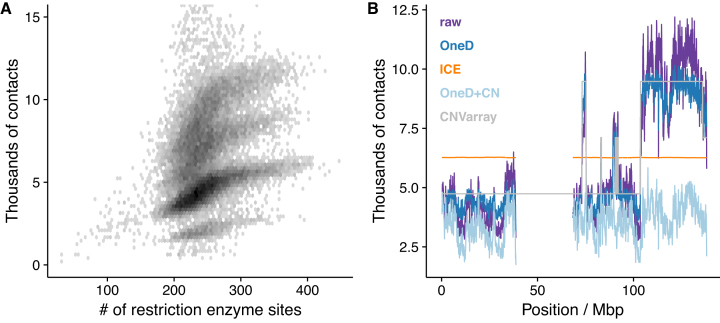
Modeling biases from the contact profile. (**A**) Nonlinear relationship between the number of restriction sites and the total number of contacts per bin in T47D. (**B**) Total number of contacts per bin on chromosome 9 of T47D. The purple line represents the raw signal, the dark blue line represents the signal after OneD bias correction, the light blue line represents the signal after OneD bias and copy number (CN) correction and the orange line represents the signal after ICE bias correction. The grey line represents the copy number estimation by independent CNV array and it has been scaled to match the figure ranges. Some regions of the long arm of chromosome 9 (the region corresponding to 60–140 Mb) are present in four copies, explaining that the signal is about twice higher than for the short arm. Both figures present data at 100 kb resolution

Given that this relationship might not be linear (see for instance Figure 1A), we allowed a smooth representation using thin plate penalized regression splines (21) in a generalized additive model (22). Thin plate regression splines are ‘isotropic’ because rotation of the covariate co-ordinate system does not change the result of smoothing and ‘low rank’ because they have far fewer coefficients than there are data points to smooth. The model can be parametrized as

\begin{eqnarray*} t_i = \sum _{j=1}^n{x_{ij}} \sim NB(\lambda _i, \theta ) \text{ and} \\ \log (\lambda _i) \propto \sum _{k}{f_k(z_{k,i})}, \end{eqnarray*}

where *x*_*ij*_ is the raw number of contacts between bins *i* and *j*, and *z*_*k, i*_ is the additive bias of genomic feature *k* in bin *i*.

The smooth functions {*f*_*k*_( · )} are estimated through the thin plate spline penalty, jointly with the negative binomial dispersion parameter θ using the the default arguments of the mgcv::s and mgcv::nb functions (22) of the R software (23).

Once the parameters of the model are determined, the estimated means {λ_*i*_} are rescaled to obtain a correction vector $\lbrace \lambda _i^{\prime }\rbrace$ that can be used to compute the corrected counts $\hat{x}_{ij}$.

(1) \begin{eqnarray*} \nonumber \lambda _i^{\prime } &= \frac{\lambda _i}{\sum _j^n{\lambda _j}/n} \\ \hat{x}_{ij} &= \frac{x_{ij}}{\sqrt{\lambda _i^{\prime }\lambda _j^{\prime }}} \end{eqnarray*}

In line with previous methods (13,15), the default features used to fit the model are the local G+C content, the read mappability and the number of restriction sites. The model and the implementation can be modified or extended with any user-provided genomic features.

### Copy number correction

Briefly, a hidden Markov model with emissions distributed as a Student’s *t* variable is fitted on the corrected total amount of contacts per bin $\hat{t}_i = \sum _{j=1}^n{\hat{x}_{ij}}$. The model consists of 8 states that correspond to 1, 2, 3, 4, 5, 6, 7 and ‘8 or more’ copies of the target bin, for a total of four emission parameters (a single position parameter for states 1–7, a position parameter for state ‘8 or more’, a single standard deviation for all the states and a single degree of freedom for all the states) and 56 transition parameters.

The model is fitted with the Baum–Welch algorithm (24) until convergence, following a previously described implementation (25). The Viterbi path is then computed and corresponds to the inferred copy number of each bin *c*_*i*_.

A correction equal to the square root of the copy number is then applied to the whole matrix. More specifically, the entry at position (*i, j*) is updated to

(2) \begin{equation*} \hat{x}_{ij}^* = \frac{\hat{x}_{ij}}{\sqrt{c_ic_j}}. \end{equation*}

### Data sources

We gathered a set of published Hi-C data (26–31) and unpublished data of different cell types and organisms (Supplementary Table S1).

The Hi-C experiments were performed in T47D breast cancer cell lines (aberrant karyotype, six samples), K562 leukemia cell lines (aberrant karyotype, eight samples), mouse primary B cells (normal karyotype, six samples) ES cells (normal karotype, seven samples), GM12878 B-lymphoblastoid cells (normal karyotype, fifty eight samples), BT474 breast cancer cell line (aberrant karyotype, one sample), MCF10 breast cancer cell line (aberrant karyotype, one sample), MCF-7 cancer cell line (aberrant karyotype, one sample) and SKBR3 breast cell lines (aberrant karyotype, one sample).

The experiments were carried out in different laboratories, following either the original Hi-C protocol (7) or the newer *in situ* version (29), and using different restriction enzymes (DpnII, HindIII, MspI, MboI and NcoI). In the figures, ‘same protocol’ means same laboratory, same Hi-C protocol and same restriction enzyme, and ‘different protocol’ means that any of the three is different.

We also used array-based copy-number segmentation of the two cell lines obtained from the COSMIC database (32) as an external reference for validation.

All data were processed through a pipeline based on TADbit (33). Briefly, after controlling the quality of FASTQ files, paired-end reads were mapped to the corresponding reference genome (hg38 or mm10) taking into account the restriction enzyme site. Non-informative contacts were removed applying the following TADbit filters: self-circle, dangling-ends, error, extra-dangling-ends, duplicated and random-breaks (for more details see (26)). In addition, the pipeline is available from the Supplementary Material published by (34).

We developed the routines contained in the dryhic R package (available at http://www.github.com/qenvio/dryhic) to efficiently create sparse representations of contact matrices and further apply *vanilla* (a single iteration of ICE), *ICE, KR* and *oneD* corrections. HiTC (35) and HiCapp (18) were used to carry out the *HiCNorm* and *caICB* corrections respectively. When not specified, the resolution of the analysis is 100 kb.

### Simulations

Simulations were based on altering four experimental Hi-C samples obtained from diploid mouse cell lines (b7fa2d8db_bfac48760, fc3e8b36a_7bf1bf374, GSM987817 and GSM862720, see Supplementary Table S1), that were performed in either B cells or ES cells, and from either CRG (Center for Genomic Regulation) or UCSD (University of California San Diego). The simulation strategy was the following: First we selected uniformly at random the amount of copy number break points (from 3 to 10). We then placed the break points along chromosomes 18 and 19 uniformly at random. Next, we assigned a copy number (2, 3, 4 or 10 with equal probability) to each segment delimited by the break points. We computed the outer product of this simulated copy number profile and multiplied it element-wise with the original contact matrices of chromosomes 18 and 19. The resulting matrices were used as input for correction methods. For each simulation (100 in total), we measured the pairwise reproducibility score defined by (36) and computed the average for pairs from the same cell type minus the average for pairs from different cell types.

### Comparison of Hi-C matrices

There is no universally accepted standard to compare Hi-C matrices. The simplest metric is the Spearman correlation applied to intra-chromosomal contacts up to a given distance (5 Mb in what follows). The second option is to measure the similarity of observed over expected contacts via the Pearson correlation up to a given distance range. Compared to the first, this metric gives more weight to changes occurring away from the diagonal. The third option is to compute a correlation per distance stratum and then obtain a stratum-adjusted correlation coefficient (SCC) as defined by (37). Finally, the ‘reproducibility score’ proposed by (36) sums the distances between the leading 20 eigenvectors of the Laplacian of the Hi-C matrix. This approach borrows the concepts of spectral clustering (38) and amounts to comparing high level features of the matrix.

We defined five data sets to measure experimental reproducibility after normalization: The first contained the samples from T47D plus two samples from K562, the second contained the samples from K562 plus two samples from T47D, the third contained all the mouse samples, the fourth contained all the GM12878 samples and the fifth contained one sample of each breast cancer cells (see Supplementary Table S1 for details). Given a set of experiments and a metric, we first computed all pairwise combinations between experiments and then classified the comparisons according to the characteristics of each pair (cell type, protocol, batch and treatment).

To measure the gain or loss of similarity upon normalization, we compared raw matrices to obtain a baseline. The differences with this baseline were estimated using a linear mixed model fitted with the lmer function of the lme4 R package (39), where the fixed effect was the normalization method and the random effect was the chromosome. To test the ability of the different methods to separate samples from different cell origin we generated receiver operating characteristic (ROC) curves using the similarity metric (e.g. reproducibility score) as the classifier score and the relative cell type (i.e. same versus different) as the binary classification. ROC curves were computed using the ROCR package (40).

## RESULTS

### Bias correction in Hi-C experiments

The principle of *OneD* is to explicitly model Hi-C biases on a single variable: the total amount of contacts for each bin of the matrix, also referred to as the *contact profile*. The reason for this choice is that the total amount of contacts is approximately proportional to the local copy number. For instance, a duplicated region in a diploid genome will show on average a 50% increase in the number of contacts. Discontinuities of the amount of contacts thus correspond to changes of the copy number.

Experimental biases affect the contact profile in a continuous but not necessarily linear way. Figure 1A shows the relationship between the amount of contacts and the number of restriction enzyme sites in T47D, a breast cancer cell line with an aberrant karyotype. Four clouds are visible. Each corresponds to sequences present in one to four copies. In all of them, the relationship flattens as the number of restriction sites increases. To capture this relationship, *OneD* fits a non-linear model between the total amount of contacts and the known sources of bias (by default the G+C content, the number of restriction sites and the mappability of the reads).

The experimental biases are estimated genome-wide and each entry of the Hi-C matrix is then corrected using equation (1). Note that the corrected contact profile is still proportional to the copy number. In cancer cell lines, the contact profile corrected by *OneD* is highly correlated to the copy number estimated from an external source (Supplementary Figure S1). Figure 1B shows the corrected contact profile along chromosome 9 of T47D, where *OneD* greatly reduces the fluctuations (dark blue line).

Optionally, *OneD* allows the user to also correct the Hi-C signal for the copy number. In this case, a hidden Markov model is fitted on the corrected contact profile in order to infer the local copy number. Each entry of the Hi-C matrix is then further corrected using equation (2), *i.e*. it is divided by the square root of the inferred copy number. In essence, this approach flattens the contact profile so that it is no longer proportional to the local copy number. The light blue line in Figure 1B shows the result of this process. After this correction, the contact profile fluctuates around a genome-wide constant value.

In what follows, we benchmarked *OneD* and *OneD* with copy number correction (*OneD+CN*) against *vanilla, ICE* (16), the *KR* algorithm (41), *caICB* (18) and the Local Genomic Features method (*HiCNorm*, 15,35). The first four methods correct biases implicitly, whereas the fifth method does it explicitly.

We used four metrics to compare Hi-C matrices (see Materials and Methods). For consistency and concision, the results based on the reproducibility score of (36) are shown in the main figures, and the results based on the other metrics are shown in the Supplementary Material.

### Aberrant karyotypes

We first benchmarked the performance of our approach on biological samples with an aberrant karyotype. A good normalization method should increase the similarity between biological replicates by reducing irrelevant experimental variance, such as batch effects, laboratory of origin and protocol variations. Likewise, a good normalization should increase the contrast between different samples to enhance the biological differences.

**Figure 2.**
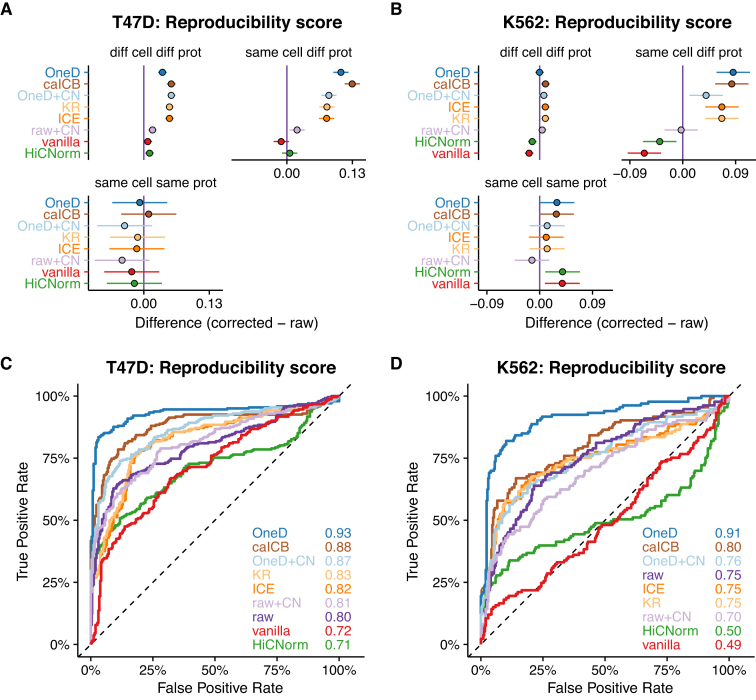
Removing biases from Hi-C on aberrant karyotypes. (**A** and **B**) Average changes compared to the raw data on the T47D and K562 data sets. The bars represent 95% confidence intervals centered on the mean difference of the reproducibility score between a given correction method and the raw data. Upper left panels: Different cell type samples processed using different protocols. Upper right panels: Same cell type samples processed using different protocols. Lower left panels: Same cell type samples processed using the same protocol. (**C** and **D**) ROC curves on the T47D and K562 sets. The areas under the curve are indicated in the bottom right corner. The color code is the same as in panels A and B.

We collected multiple Hi-C data sets obtained from T47D and K562 cells, both with aberrant karyotypes. The first pool, referred to as the T47D data set contained six T47D samples and two K562 samples, the second, referred to as the K562 data set contained eight K562 samples and two T47D samples. We compared matrices before and after normalization by different methods (see Materials and Methods, distributions shown in Supplementary Figures S2 and S3). This gave an indication of the impact of a given normalization method on the biological variation (differences between samples from T47D and K562) and on the technical variation (differences among samples from the same cell type). The results are summarized in Figure 2 and Supplementary Table S3.

On the T47D data set, the methods that increased the reproducibility between identical cell types also increased it between different cell types (Figure 2A), meaning that none of them enhanced exclusively the biological signal. However, the difference was most marked for *OneD* and *caICB*. On the K562 data set (Figure 2B), both methods increased the reproducibility between identical cell types but not between different cell types, which is the desired behavior of a correction method. On both data sets, *OneD+CN, ICE* and *KR* gave inferior results, while *raw+CN* (copy number correction alone), *HiCNorm* and *vanilla* were not competitive. The conclusions were unchanged when using other metrics to compare Hi-C matrices (Supplementary Figures S4–S6).

An important application of normalization methods in experimental setups is to identify outliers. We thus investigated the capacity of the different methods to help identify the samples from the other cell type spiked in the data set. We interpreted the pairwise comparison scores as classifier scores and summarized the results with a ROC curve (Figure 2C and D). *OneD* had the largest area under the curve in both tests, and the corresponding ROC curve was above the others throughout. Using other metrics to compare Hi-C matrices yielded similar results (Supplementary Figures S4–S6).

Taken together, these results show that *OneD* enhances the biological variation and reduces the noise on samples with an aberrant karyotype.

### Normal karyotypes

Does the performance of *OneD* on aberrant karyotypes come at the cost of decreased performance on normal karyotypes? To address this question, we collected data from mouse B cells and embryonic stem (ES) cells, both with a normal karyotype. The cell types were pooled in almost equal proportions (see Supplementary Table S1) and we performed the same tests as above using the same metrics (distributions shown in Supplementary Figure S7).

**Figure 3.**
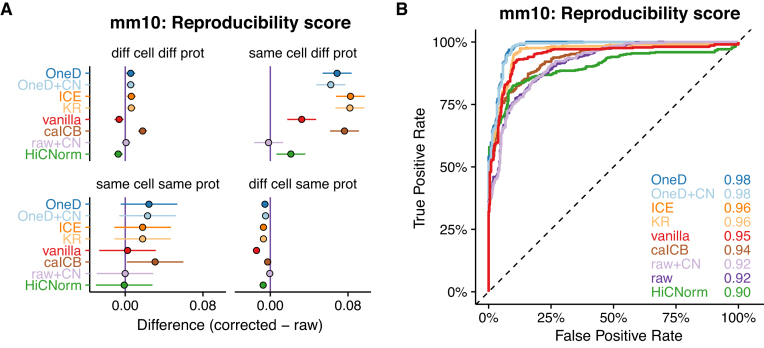
Removing biases from Hi-C on normal karyotypes. (**A**) Average changes compared to the raw data on the mouse data set. Upper left panel: Different cell type samples processed using different protocols. Upper right panel: Same cell type samples processed using different protocols. Lower left panel: Same cell type samples processed using the same protocol. Lower right panel: Different cell type samples processed using the same protocol. (**B**) ROC curves on the mouse data set. The legend and color code are as in Figure 2.

In this test, the reproducibility between different cell types was barely affected (Figure 3A). All the methods increased the reproducibility between identical cell types (except one, see below), but only when the experimental conditions were different. This means that all the methods were able to remove some technical biases. *ICE* and *KR* showed the strongest increase in reproducibility, but *OneD, OneD+CN*, and *caICB* had competitive performance. *vanilla* and *HiCNorm* were less competitive, and *raw+CN* (copy number correction alone) performed poorly, because it is equivalent to no normalization on diploid cells. Other metrics gave similar results (Supplementary Figures S8–S10, Supplementary Table S3).

As above, we used the reproducibility score for classification and compared the tools with a ROC curve (Figure 3B). The performance of all the tools were high and less variable than for aneuploid cell lines. *OneD* achieved the highest area under the curve on this problem, but with a small margin over *KR* and *ICE*. The performance of *OneD+CN* was the same as that of *OneD* because copy number correction has no effect on diploid cell lines. Using other metrics to compare matrices gave similar results (Supplementary Figures S8–S10).

We took advantage of the wide range of protocols used in the GM12878 set to further investigate the performance of each method. The differences between methods were minor and *OneD* showed the greatest improvement in reproducibility (Supplementary Figure S11).

Taken together, these results indicate that *OneD* performs as well as the best normalization methods, even with normal karyotypes.

### Copy number correction

The results so far suggest that the copy number correction lowers the performance of *OneD*. A possible limitation in Hi-C is the absence of ground truth. We thus used simulations to generate matrices with defined karyotypic aberrations (see Materials and Methods) and tested the capacity of the correction methods to distinguish different cell types (Supplementary Figures S12 and S13). On this test, *HiCNorm* and *OneD* performed best. In comparison, *OneD+CN* performed less well, suggesting that correcting the copy number attenuates the biological signal. This is corroborated by the fact that the copy number correction alone has a lower performance than no correction at all. Thus, copy number correction can indeed have side effects that lower the biological signal.

However, those conclusions are based on data sets where the karyotype is uniform. One sometimes needs to compare samples from cells with different karyotype, for instance in cancer samples where the karyotypes may change through time. In such conditions, copy number correction can remove a feature that is considered irrelevant in order to highlight other biological differences.

**Figure 4.**
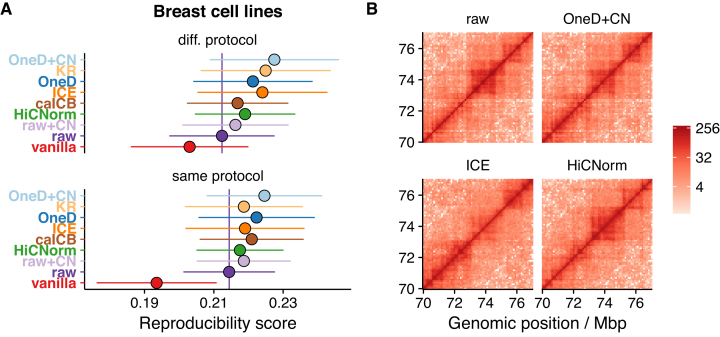
Copy number correction. (**A**) Removing biases from Hi-C on breast cancer cells. The reproducibility score between samples from the same breast cancer cell type was computed after normalization. The vertical bar shows the reproducibility score of the raw data. Shown are comparisons for different (top) or identical (bottom) experimental conditions. (**B**) Detail of a Hi-C matrix normalized with different methods. The central portion has an increased copy number, which affects normalization. *ICE* fades it away, *HiCNorm* enhances it and *OneD* reduces the signal by about half.

To test this idea, we collected a Hi-C data set from breast cancer cell lines and measured the reproducibility score between identical cell types after normalization with the different tools. In this case, we found that *OneD+CN* outperformed *OneD* and gave the highest overall reproducibility (Figure 4A). Even though the variability is high in this case, this suggests that the karyotypes of these cells have diverged, so that the copy number correction performed by *OneD+CN* increased the reproducibility between samples from the same cell type.

Copy number correction is also useful to give euploid-equivalent representations of samples from aberrant karyotypes. Figure 4B shows an example where a region at the center of the matrix is duplicated. *ICE* overcompensated the original bias and faded the signal almost entirely. Concomitantly, the signal at the bottom left of the matrix was enhanced and showed a structure that was not visible in the raw data. On the contrary, *HiCNorm* strengthened the central region and the diagonal. *OneD+CN* reduced the level of the central portion by a factor 2 approximately, but did not otherwise distort the main features of the region.

Besides matrix correction, copy number estimation could have a potential stand-alone utility. For instance, we applied *OneD+CN* to a T47D sample (dc3a1e069_51720e9cf) at 10 kb and compared the correlation with the array-based copy number. The correlation increased from 0.80 for the raw totals, to 0.88 for the *OneD* corrected totals to 0.92 for the estimated CN. We provide the table of the estimated start and end points as Supplementary Table S2.

In summary, *OneD+CN* can improve the reproducibility of samples with unstable karyotypes, and it can be used to obtain an euploid-equivalent normalized matrix.

### Capture Hi-C

Matrix-balancing methods (here *ICE, vanilla* and *KR*) are specifically tailored for Hi-C because it is the dominant 3C-derived technology. As a consequence, their performance is typically lower when they are used for variants such as Capture Hi-C (42). In this case, the contacts with one or more regions of interest are enriched through direct purification, essentially erasing all the other contacts from the Hi-C matrix. The lack of homogeneity of the signal in such technologies makes normalization particularly challenging.

**Figure 5.**
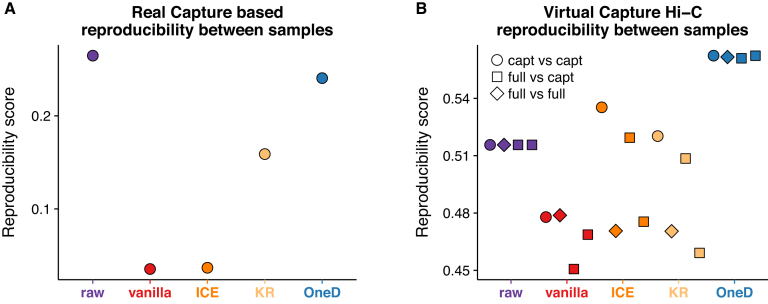
Bias removal on capture Hi-C data. (**A**) Performance on the Capture Hi-C data. Two replicates centered on a 6 Mb domain on chromosome 6 in T47D cells were normalized by the indicated tools and their reproducibility score was computed. (**B**) Performance on virtual Capture Hi-C data. Two Hi-C experiments were post-processed to produce an output similar to the Capture Hi-C experiment depicted in panel A. The signal outside the domain on chromosome 6 was removed and the data were normalized (capt), or the data were normalized before removing the signal outside the domain on chromosome 6 (full). Four matrices per method were generated and the reproducibility score between the pairs from different replicates were computed. Note that the scores are identical for the raw data because in this case the matrices of the same replicate are identical.

We reasoned that *OneD* should handle such cases gracefully, because the signal is formally equivalent to an aberrant karyotype where most of the genome is present in zero copy. We thus evaluated the performance of *OneD* on a Capture Hi-C data set at 5 kb resolution centered on a 6 Mb domain of chromosome 6 in T47D cells. We found that the reproducibility score between replicates was higher after normalization with *OneD* than with matrix-balancing methods (Figure 5A and Supplementary Figure S14). The performance of *OneD* was only slightly lower than the raw data, suggesting that it barely disturbs the biological signal. Other tools were not included because they had prohibitive run time.

To gain additional insight, we also set up a ‘virtual Capture Hi-C’ data set by removing the signal outside the same region of chromosome 6 from Hi-C experiments performed in the same cell type. In this setup, we could restrict the data and then normalize, or normalize and then restrict the data. This allowed us to probe how sensitive the methods are to the shape of the input data. Here we found that *OneD* significantly outperformed the other methods: it featured the highest reproducibility score between replicates with practically no influence from the shape of the data (Figure 5B). Also, in this case, *OneD* enhanced the reproducibility between replicates compared to the raw data. This confirms the view that capture methods are a natural framework for the statistical model underlying *OneD*. In comparison, matrix-balancing methods showed lower reproducibility between replicates, together with more variability due to the shape of the data.

In conclusion, *OneD* is suitable for removing experimental biases from data acquired with Capture Hi-C.

### Speed

Finally, we compared the computational speed of the different normalization methods. *vanilla* and *ICE* are broadly used because of their conceptual simplicity, ease of use and speed (16). This is even more significant as current explicit methods (35) are much slower in comparison.

**Figure 6.**
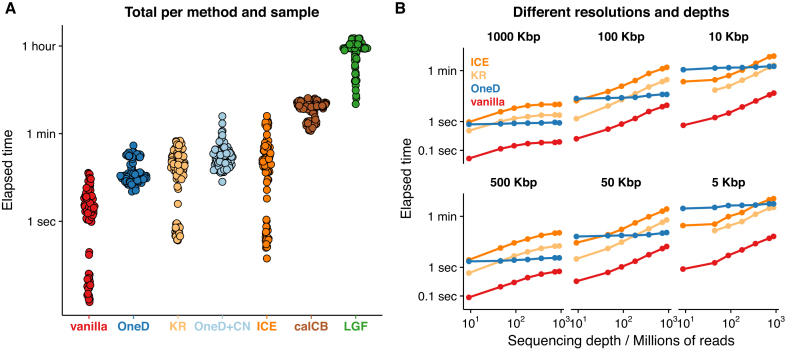
Computing time of the bias correction methods. (**A**) Total time to process samples listed in Supplementary Table S1 at 100 kb resolution. Each dot corresponds to a sample. (**B**) Total time to process sample HIC070 at the indicated bin size and sequencing depths. Note the logarithmic scale on the y-axis on both panels.

*OneD* corrects a single variable instead of the whole matrix, and thus estimates the model parameters much faster than previous explicit methods. We measured the running time of the different tools on a 3.3 GHz machine with 62GB RAM, always using the default parameters. Figure 6A shows the running times of the different methods on all the samples shown in Supplementary Table S1 at 100 kb resolution. The fastest method was *vanilla* and the slowest *HiCNorm*, with an over 100-fold span between the two.

On average, *OneD* was the second fastest method and it always ran in <1 min in the conditions of the benchmark. The copy number correction increased the running time, but it remained under 1 min in general. Throughout this benchmark, the memory footprint of *OneD* was less than 300MB. Interestingly, the running time of *OneD* was much more homogeneous than that of the other methods. The reason is that the size of the regression problem to be solved by the mgcv package is always the same at fixed resolution.

To better understand these results, we also investigated the influence of bin size and sequencing depth on the running time. We performed a benchmark on the sample with highest sequencing depth in the data set (HIC070, see Supplementary Table S1) at different bin sizes while downsampling the contact matrices to mimic lower depths. On this test, we included only the methods competing with *OneD* in terms of speed, *i.e*. the matrix-balancing methods. Figure 6B shows that the running time of *OneD* does not depend on the sequencing depth. Strikingly, *OneD* also gives nearly identical results at very different sequencing depths (Supplementary Figure S15). *vanilla* was the overall fastest method and *OneD* was usually faster than *ICE* and *KR* at high but not at low sequencing depth. At low resolution (100 kb) the speed advantage of *OneD* appeared from low sequencing depth, but at high resolution resolution (5 kb) it appeared only at high sequencing depth.

Taken together, these results show that *OneD* is competitive in terms of speed. It is particularly adapted to projects where the sequencing depth is high, as the running time is essentially not affected.

## DISCUSSION

Here we introduced *OneD*, a fast computational method to normalize Hi-C matrices. *OneD* was developed ground up to address the need to normalize data from biological samples with aberrant karyotypes, but it applies seamlessly to the case of normal karyotypes. We showed that *OneD* performs significantly better than other methods when the cells present karyotypic aberrations (Figure 2), and that it performs equally well as the best methods on euploid genomes (Figure 3). We also showed that *OneD* is approximately as fast as *ICE* (Figure 6), which makes it competitive from the point of view of computational speed.

The originality of *OneD* lies in the estimation from a single variable of the explicit biases with a Negative Binomial model and of the copy number. This allows greater running speed, while preserving a good performance on samples with karyotypic aberrations. One of the reasons why *OneD* is able to better highlight the biological distinctions between samples is that it only corrects the copy number if specifically requested by the user. The impact of copy number variations on normalization is rather opaque, especially if they are treated as implicit biases (Figure 4B). Treating them as explicit biases with optional removal seems to be an overall safer strategy. In homogeneous data sets, correcting for the copy number can blur the biological signal, but when karyotypes are variable or unstable, it may increase the reproducibility (Figure 4A). If the purpose is to remove the effect of copy number, then OneD+CN outperforms a karyotype correction method such as caICB because it allows for more flexible types of aberrations. On the other hand, if the purpose is to improve the reproducibility among samples of the same type, correcting the copy number removes part of the signal, and therefore blurs the distinction between experiments (Figures 2 and 3).

This raises the question whether variations of the copy number constitute a biological signal or an artifact. If the biological sample contains karytoypic aberrations, then its genome is grossly different from the reference genome, which makes signal correction very challenging. The proper approach would be to use the actual genome of the biological sample as a reference to construct the contact map. However, this strategy is presently unfeasible because assembling mammalian genomes is still a hard problem.

Depending on the intention of the user, the effect of the copy number should either be kept or removed. This is why *OneD* does not perform the correction by default, but allows the user to obtain a euploid-equivalent Hi-C map computed from a hidden Markov model. The resulting matrices have a near constant amount of contacts per bin, but the artifacts caused by the mismatch between the genome of the sample and the reference genome are still present (for instance, the artifacts caused by large scale inversions are not changed in any way).

## CONCLUSION

Overall, *OneD* constitutes a novel computational approach to normalize Hi-C matrices. If the karyotype of the sample is aberrant, it enhances the biological variation. If not, it performs at least equally well as other methods in terms of quality and of computational speed.

## AVAILABILITY

The *OneD* R package and the data used here are available at http://www.github.com/qenvio/dryhic.

## Supplementary Material

Supplementary Data
