# Supplementary material for: OneD: increasing reproducibility of Hi-C samples with abnormal karyotypes
Source: Nucleic Acids Res. 2018 Jan 31;46(8):e49. doi: 10.1093/nar/gky064 (PMC5934634; doi:10.1093/nar/gky064)
Supplement: Supplementary Data [file gky064_supp.zip › nar-01720-met-n-2017 -File012.pdf]

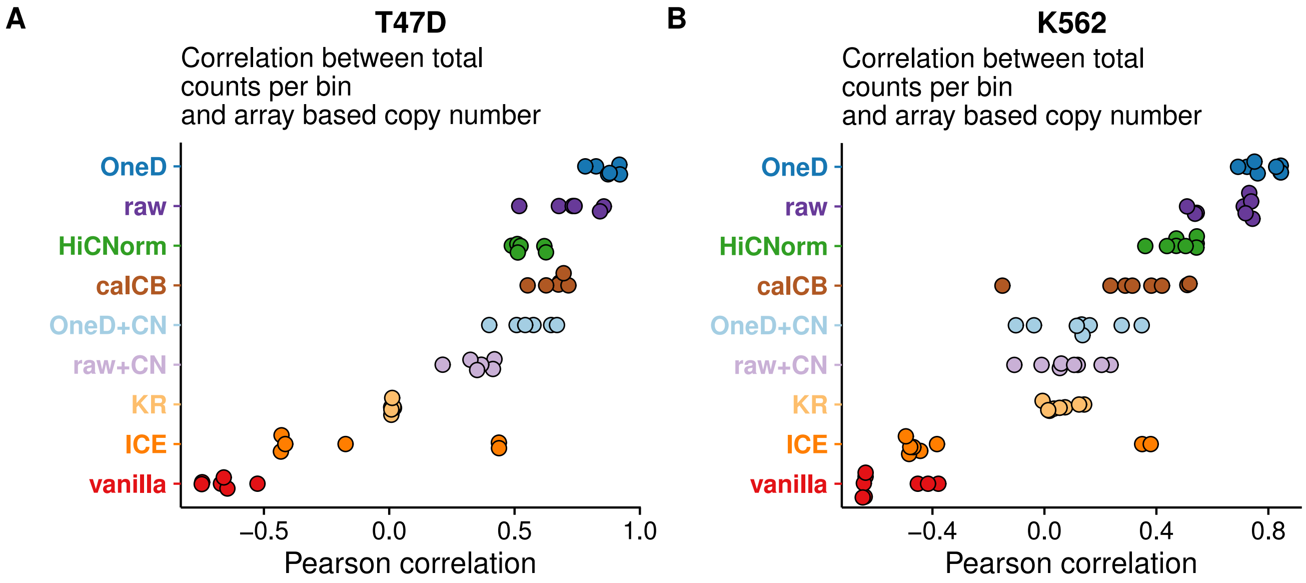

Figure S1: Pearson correlation between contact profiles and independent copy number estimation from the COSMIC data base (Forbes *et al.*, 2010). A. T47D breast cancer cell line (6 samples). B. K562 leukemia cell line (8 samples). Each dot represents a sample normalized by a different tool. The data normalized by *OneD* correlates better with the copy number than the unnormalized data.

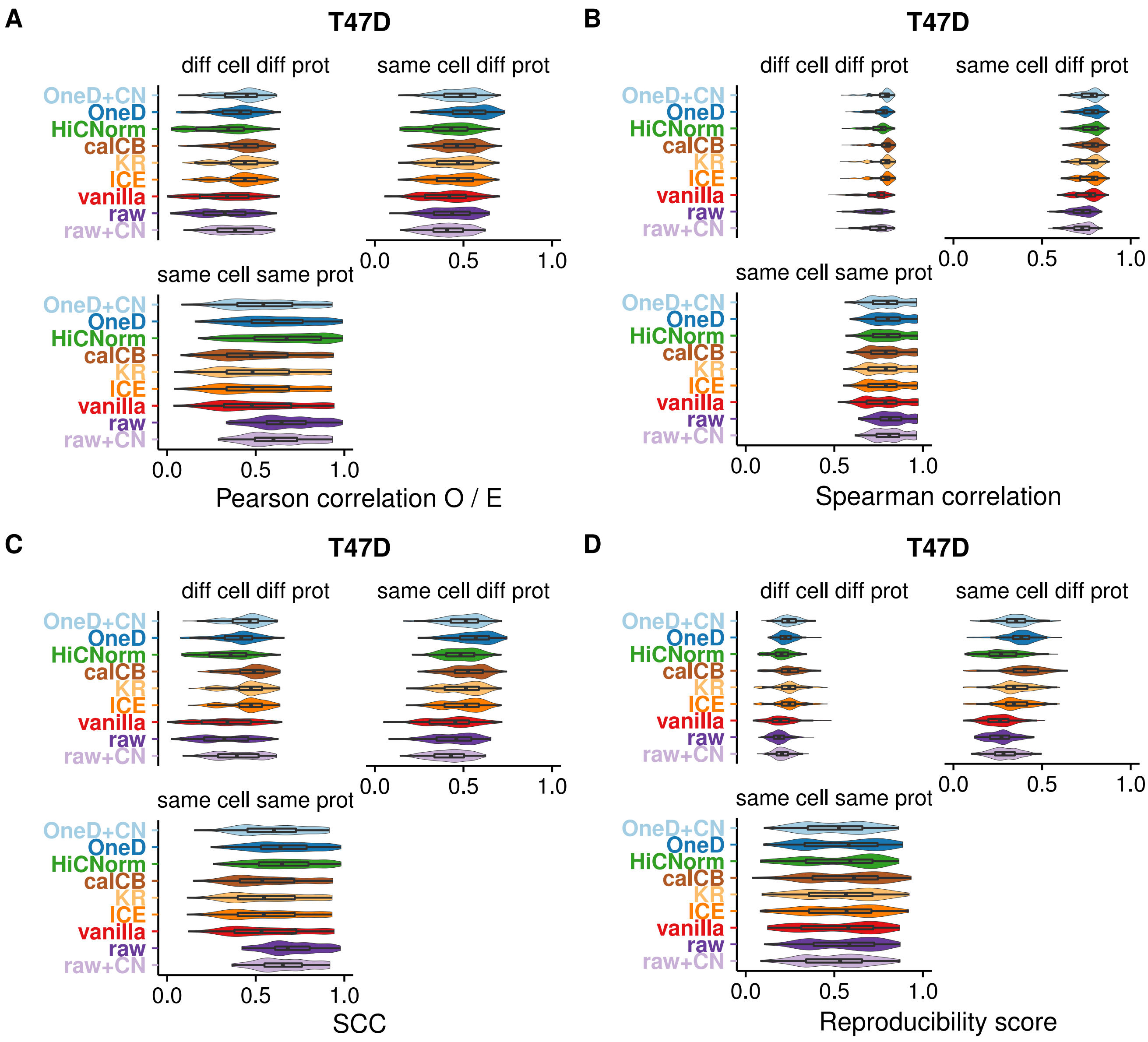

Figure S2: Distribution of the pair-wise comparisons between the samples included in the T47D set. X axis: similarity metric. Y axis: correction method. Each panel groups pairs of samples with the corresponding characteristics (in terms of cell type and protocol). A. Pearson correlation between observed over expected counts. B. Spearman correlation between observed counts. C. Stratum-adjusted correlation coefficient (SCC) between observed counts. D. Reproducibility score of observed counts.

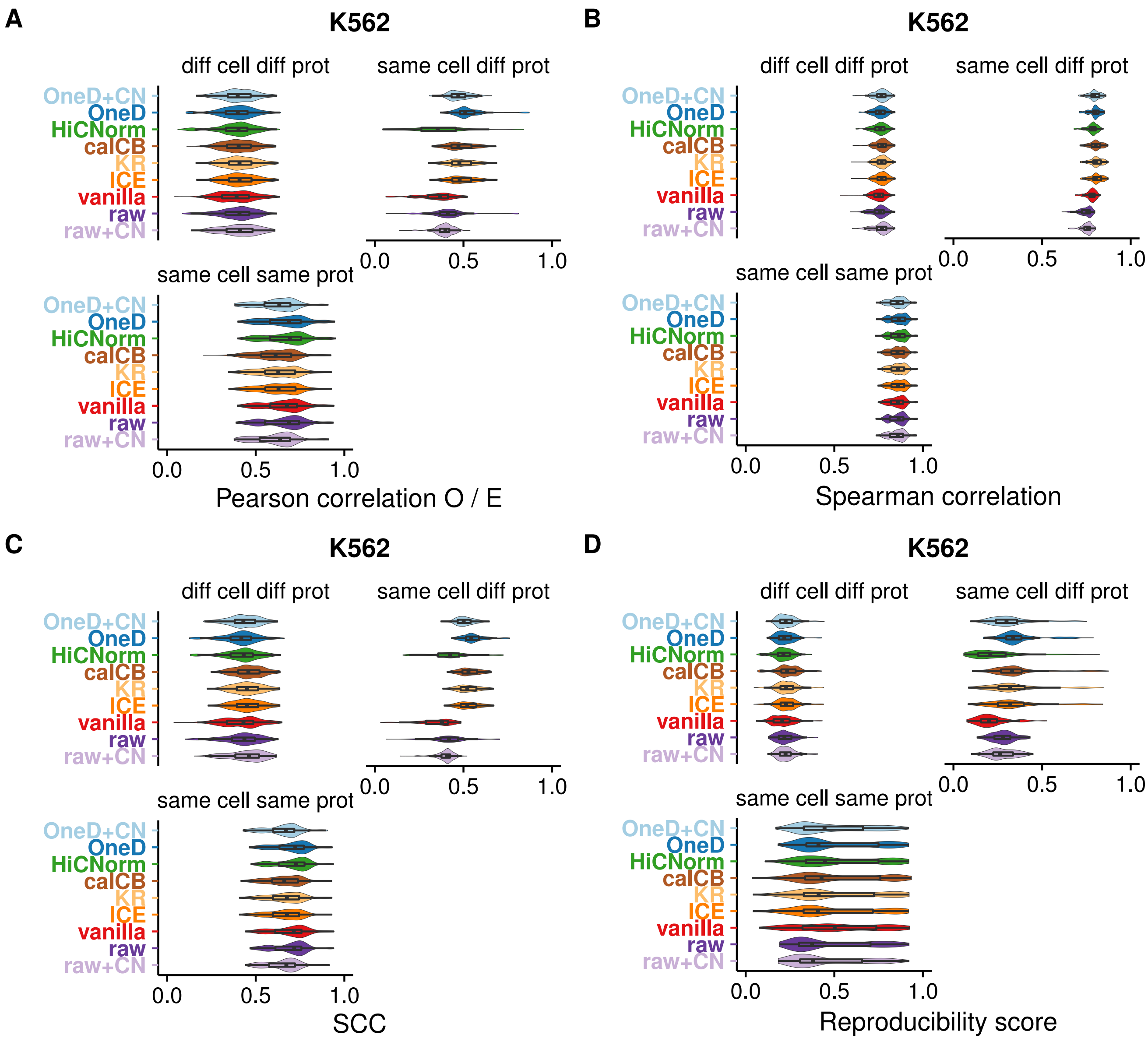

Figure S3: Distribution of the pair-wise comparisons between the samples included in the K562 set. X axis: similarity metric. Y axis: correction method. Each panel groups pairs of samples with the corresponding characteristics (in terms of cell type and protocol). A. Pearson correlation between observed over expected counts. B. Spearman correlation between observed counts. C. Stratum-adjusted correlation coefficient (SCC) between observed counts. D. Reproducibility score of observed counts.

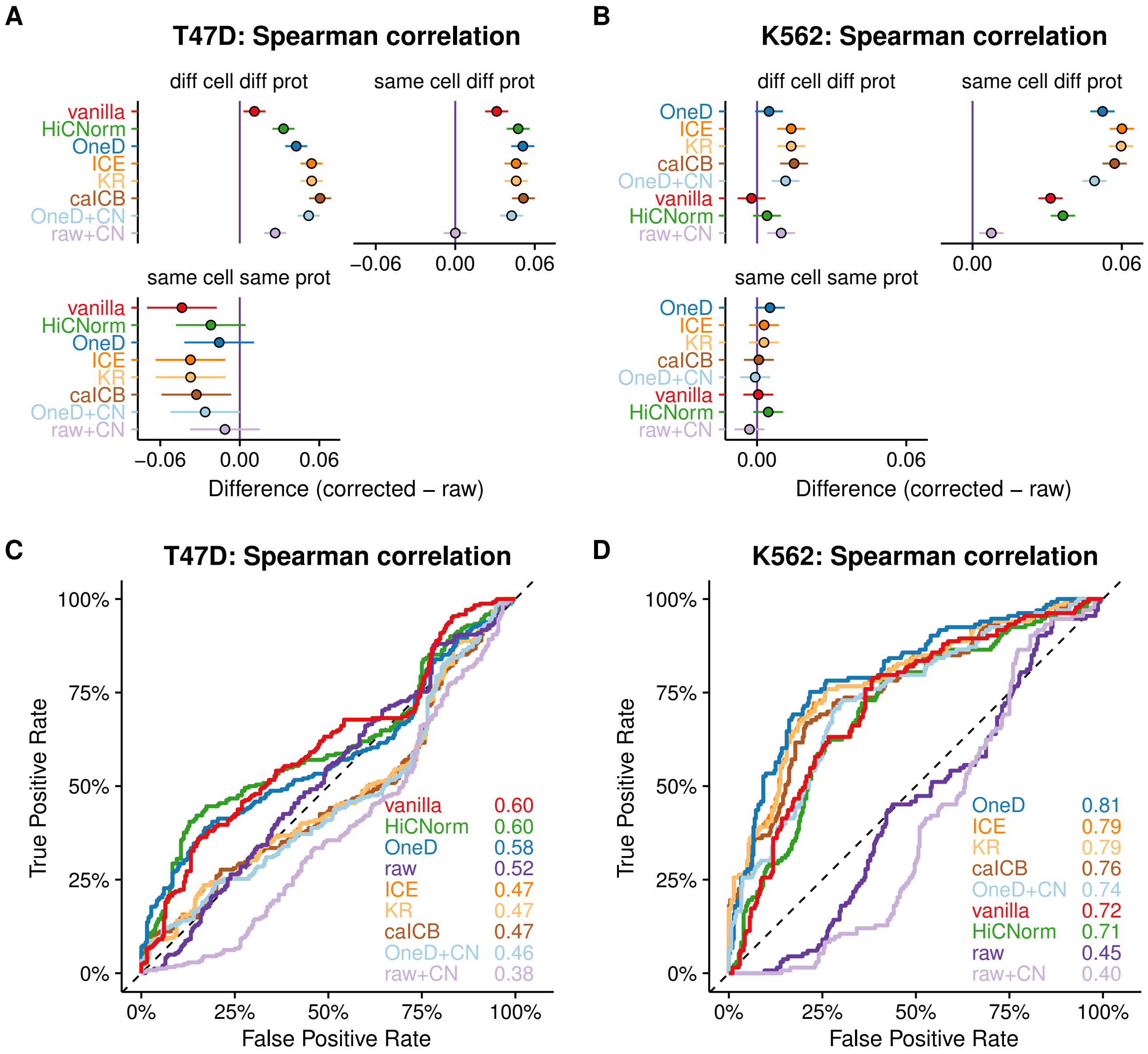

Figure S4: Removing biases from Hi-C on aberrant karyotypes (Spearman correlation). A and B. Average changes compared to the raw data on the T47D and K562 data sets. The bars represent 95% confidence intervals centered on the mean difference of the Spearman correlation between a given correction method and the raw data. C and D. ROC curves on the T47D and K562 sets. The areas under the curve are indicated in the bottom right corner. The color code is the same as in panels A and B.

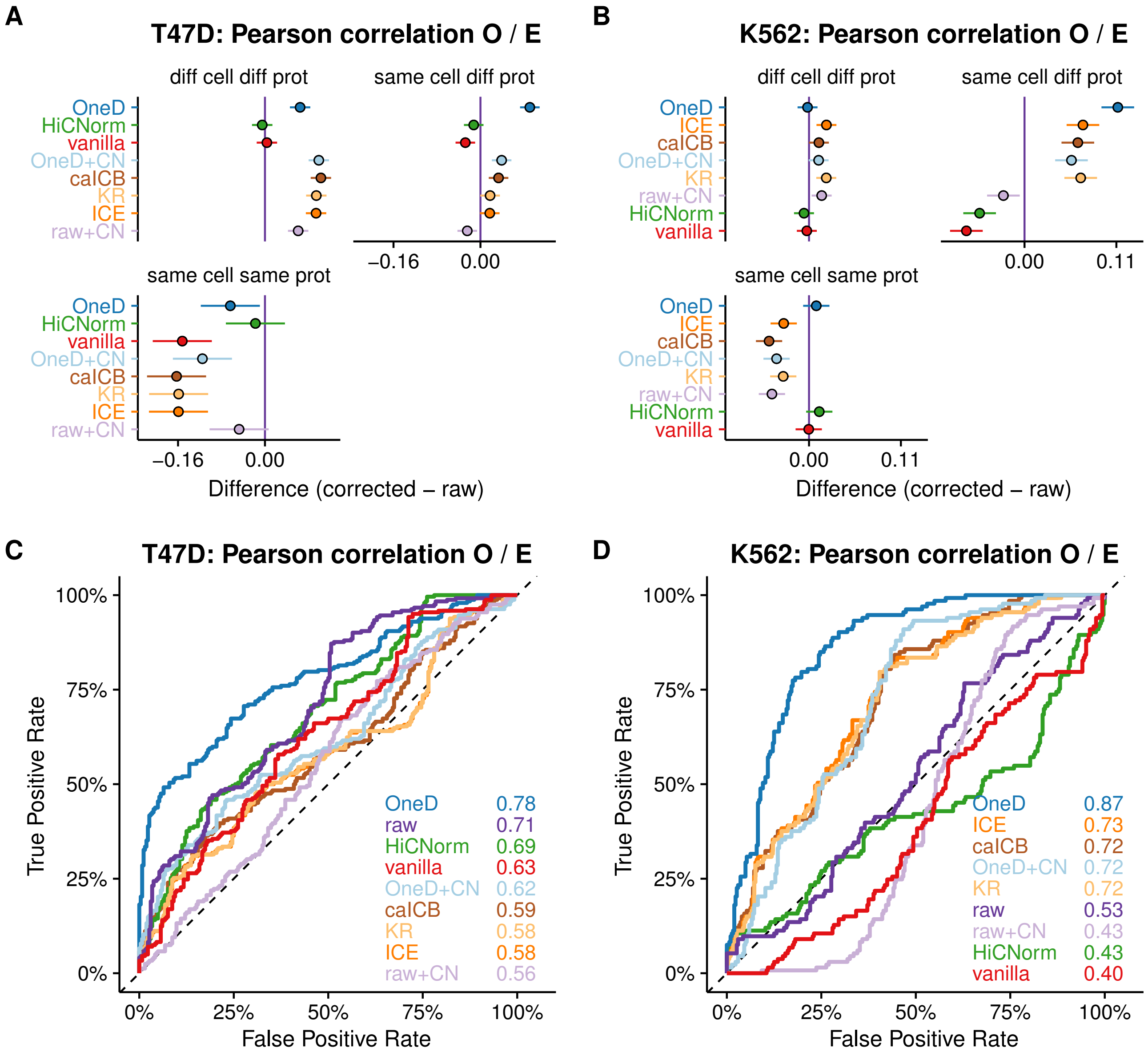

Figure S5: Removing biases from Hi-C on aberrant karyotypes (Pearson correlation). A and B. Average changes compared to the raw data on the T47D and K562 data sets. The bars represent 95% confidence intervals centered on the mean difference of the Pearson correlation of the observed over expected counts between a given correction method and the raw data. C and D. ROC curves on the T47D and K562 sets. The areas under the curve are indicated in the bottom right corner. The color code is the same as in panels A and B.

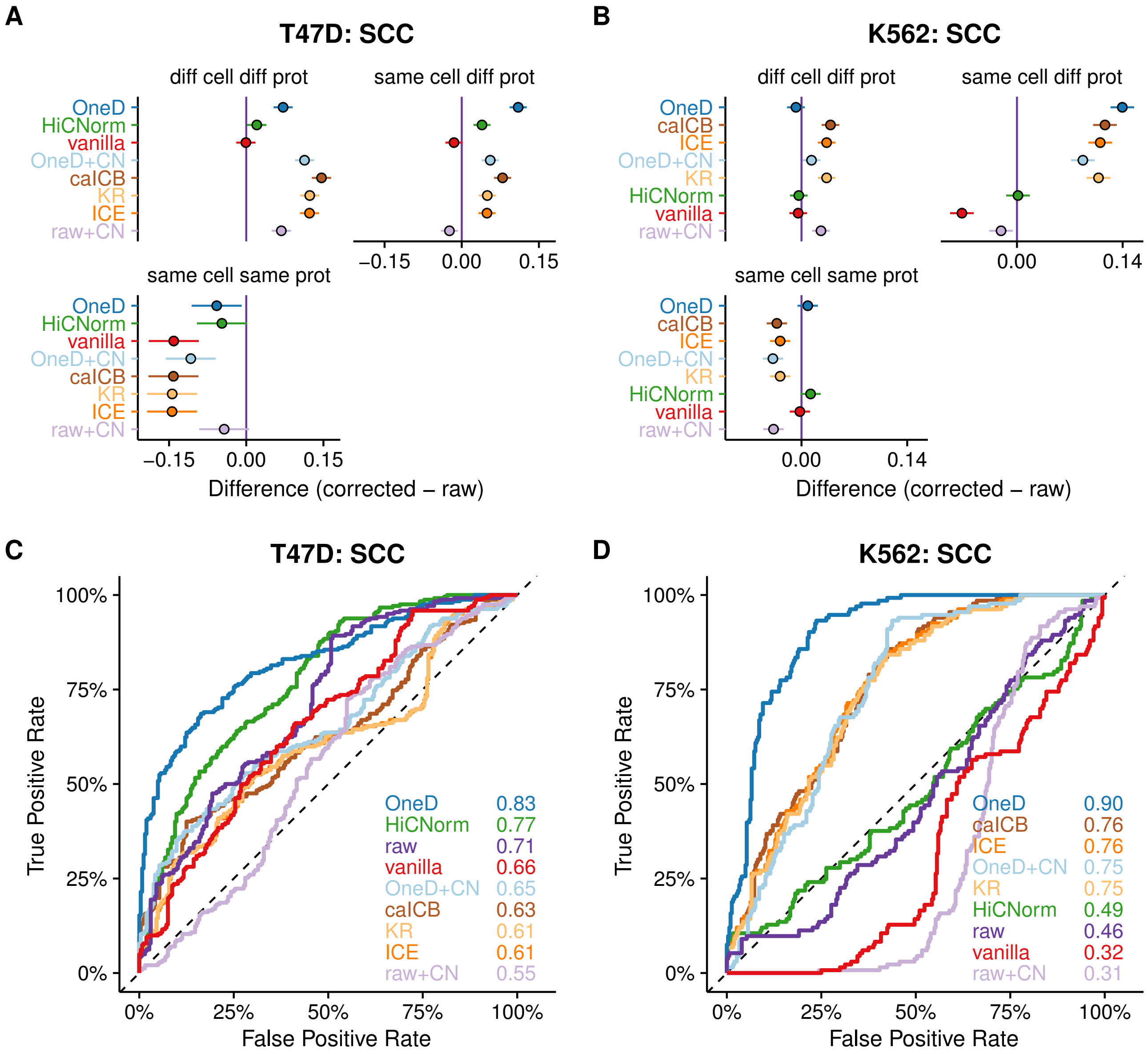

Figure S6: Removing biases from Hi-C on aberrant karyotypes (SCC). A and B. Average changes compared to the raw data on the T47D and K562 data sets. The bars represent 95% confidence intervals centered on the mean difference of the stratum-adjusted correlation coefficient (SCC) between a given correction method and the raw data. C and D. ROC curves on the T47D and K562 sets. The areas under the curve are indicated in the bottom right corner. The color code is the same as in panels A and B.

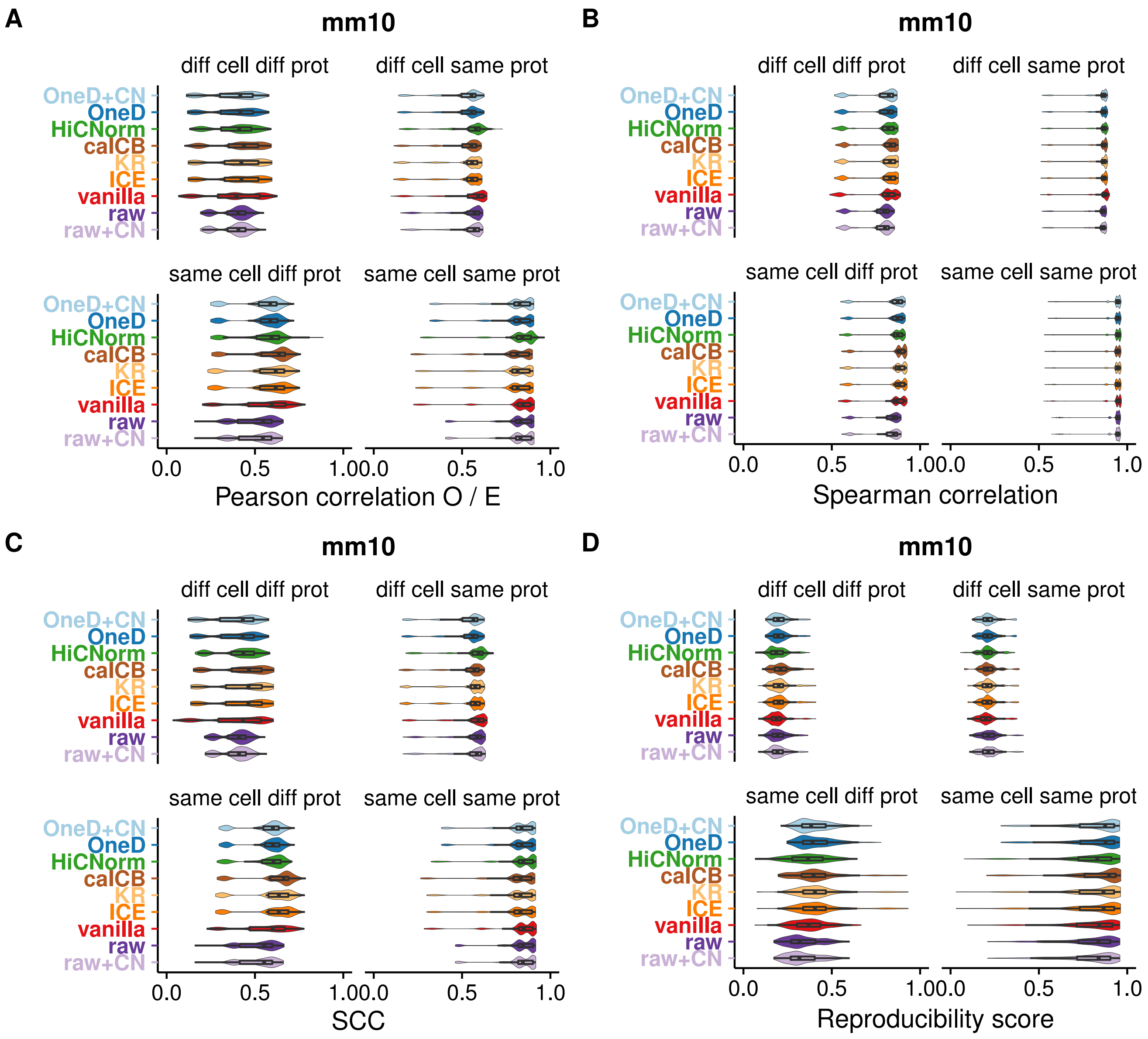

Figure S7: Distribution of the pair-wise comparisons between the samples included in the mm10 data set. X axis: similarity metric. Y axis: correction method. Each panel groups pairs of samples with the corresponding characteristics (in terms of cell type and protocol). A. Pearson correlation between observed over expected counts. B. Spearman correlation between observed counts. C. Stratum-adjusted correlation coefficient (SCC) between observed counts. D. Reproducibility score of observed counts.

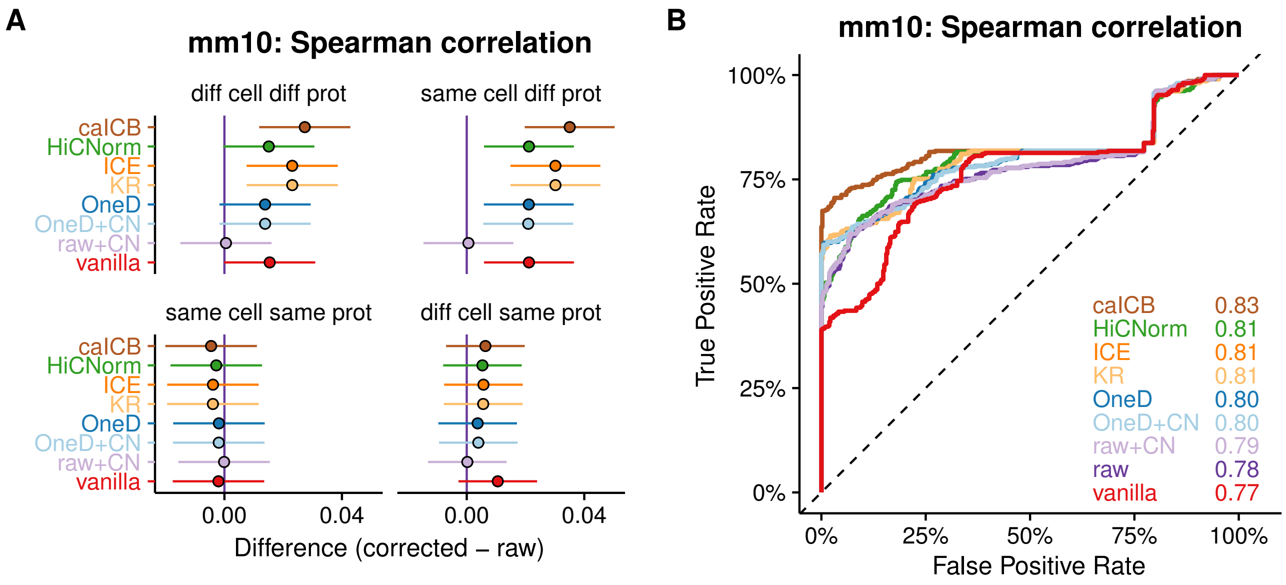

Figure S8: Removing biases from Hi-C on normal karyotypes (Spearman correlation). A. Average changes compared to the raw data on the mm10 data set. The bars represent 95% confidence intervals centered on the mean difference of the Spearman correlation between a given correction method and the raw data. B. ROC curves on the mm10 data set. The areas under the curve are indicated in the bottom right corner. The color code is the same as in panel A.

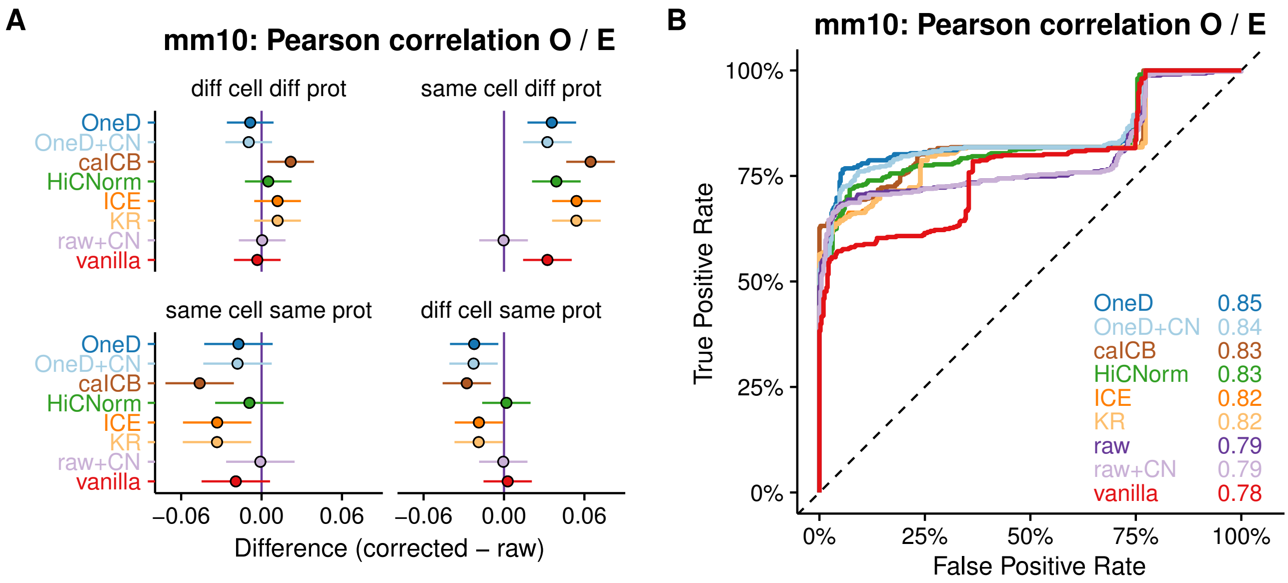

Figure S9: Removing biases from Hi-C on normal karyotypes (Pearson correlation). A. Average changes compared to the raw data on the mm10 data set. The bars represent 95% confidence intervals centered on the mean difference of the Pearson correlation of the observed over expected counts between a given correction method and the raw data. B. ROC curves on the mm10 data set. The areas under the curve are indicated in the bottom right corner. The color code is the same as in panel A.

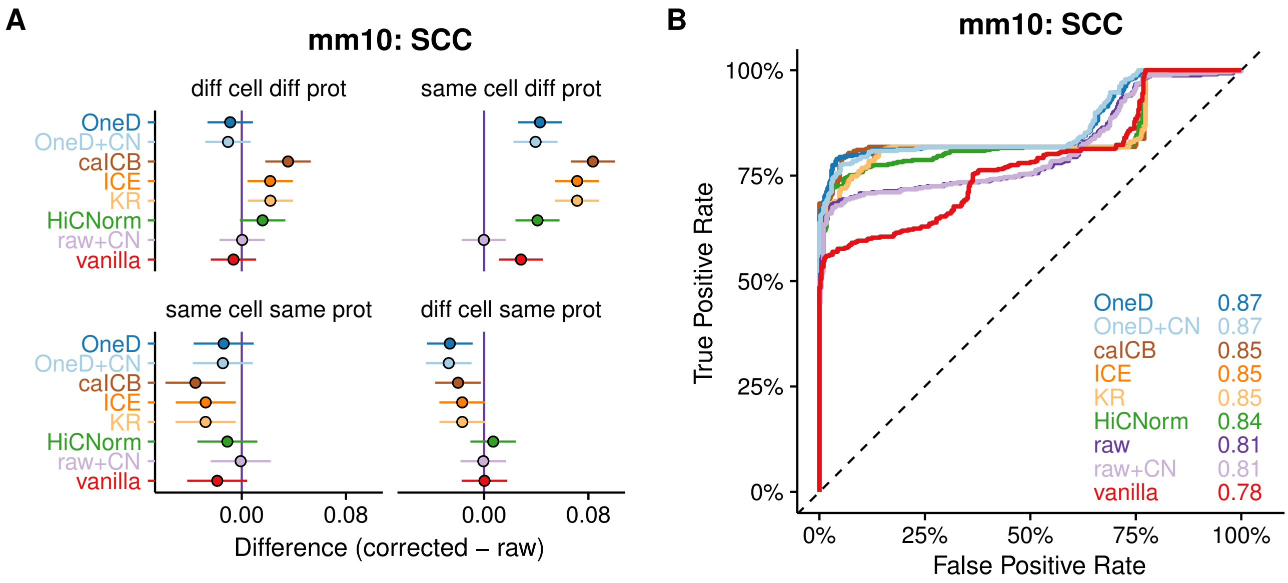

Figure S10: Removing biases from Hi-C on normal karyotypes (SCC). A. Average changes compared to the raw data on the mm10 data set. The bars represent 95% confidence intervals centered on the mean difference of the stratum-adjusted correlation coefficient (SCC) between a given correction method and the raw data. B. ROC curves on the mm10 data set. The areas under the curve are indicated in the bottom right corner. The color code is the same as in panel A.

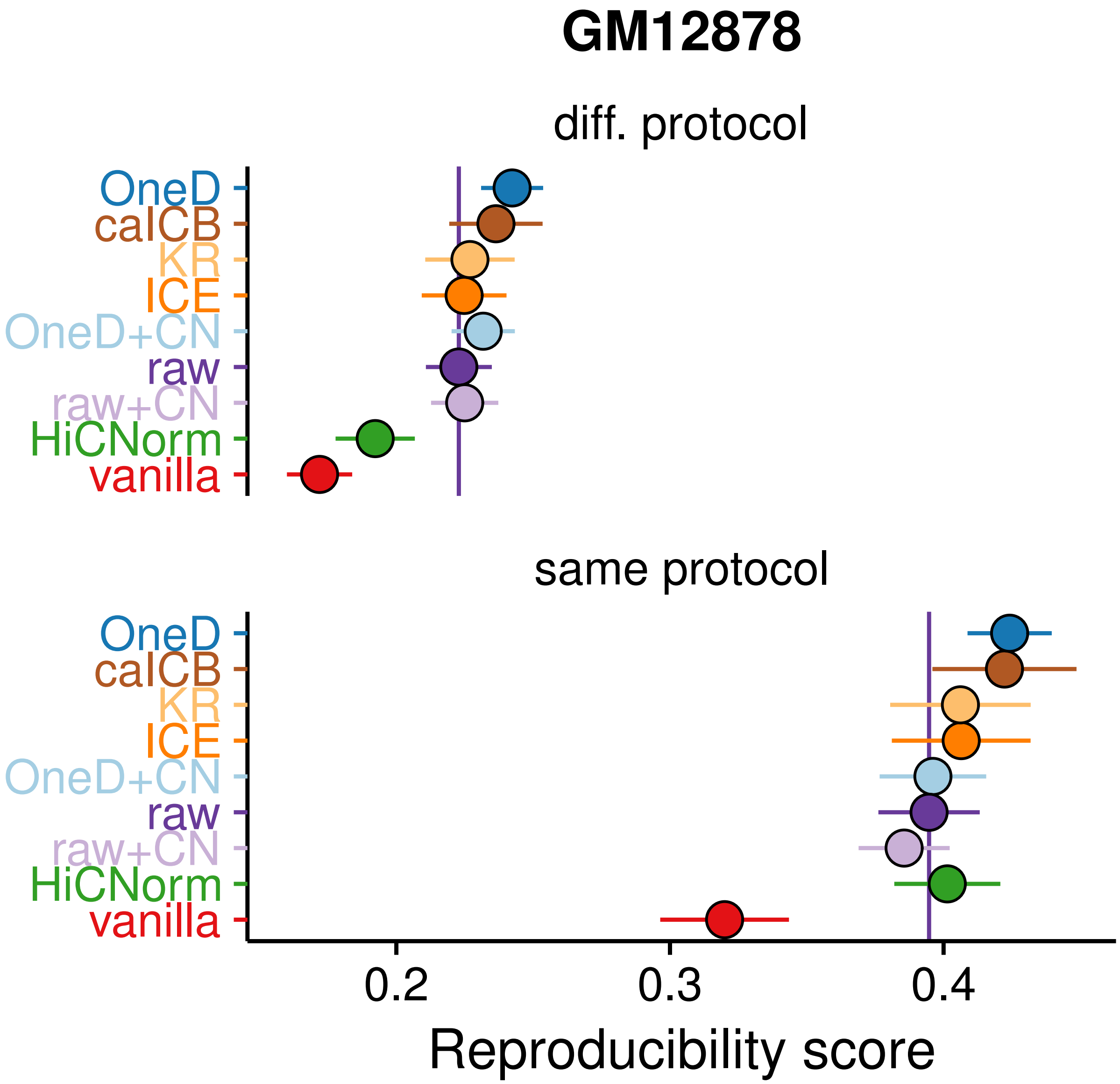

Figure S11: Removing biases from Hi-C on GM12878 samples. X axis: reproducibility score. Y axis: correction method. The bars represent 95% confidence intervals centered on the mean of the reproducibility score of each correction method. The vertical dark purple line marks the reproducibility score for the uncorrected data (raw). Upper panel: pairs of samples processed using different protocols. Lower panel: pairs of samples processed using the same protocol.

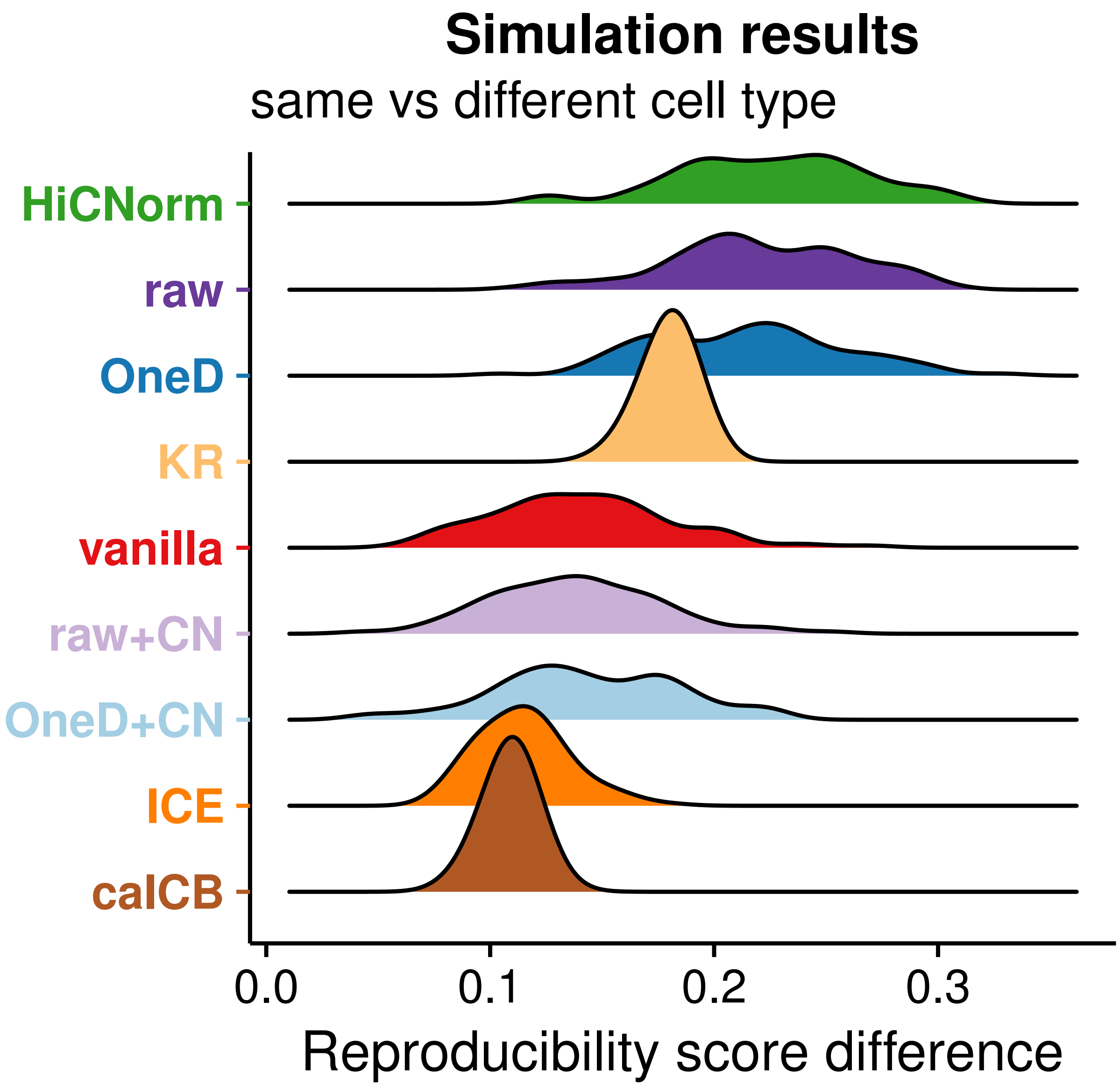

Figure S12: Distribution of the simulation results. X axis: Difference in reproducibility score between pairs of samples of the same cell type and pairs of samples of different cell type. Y axis: correction method

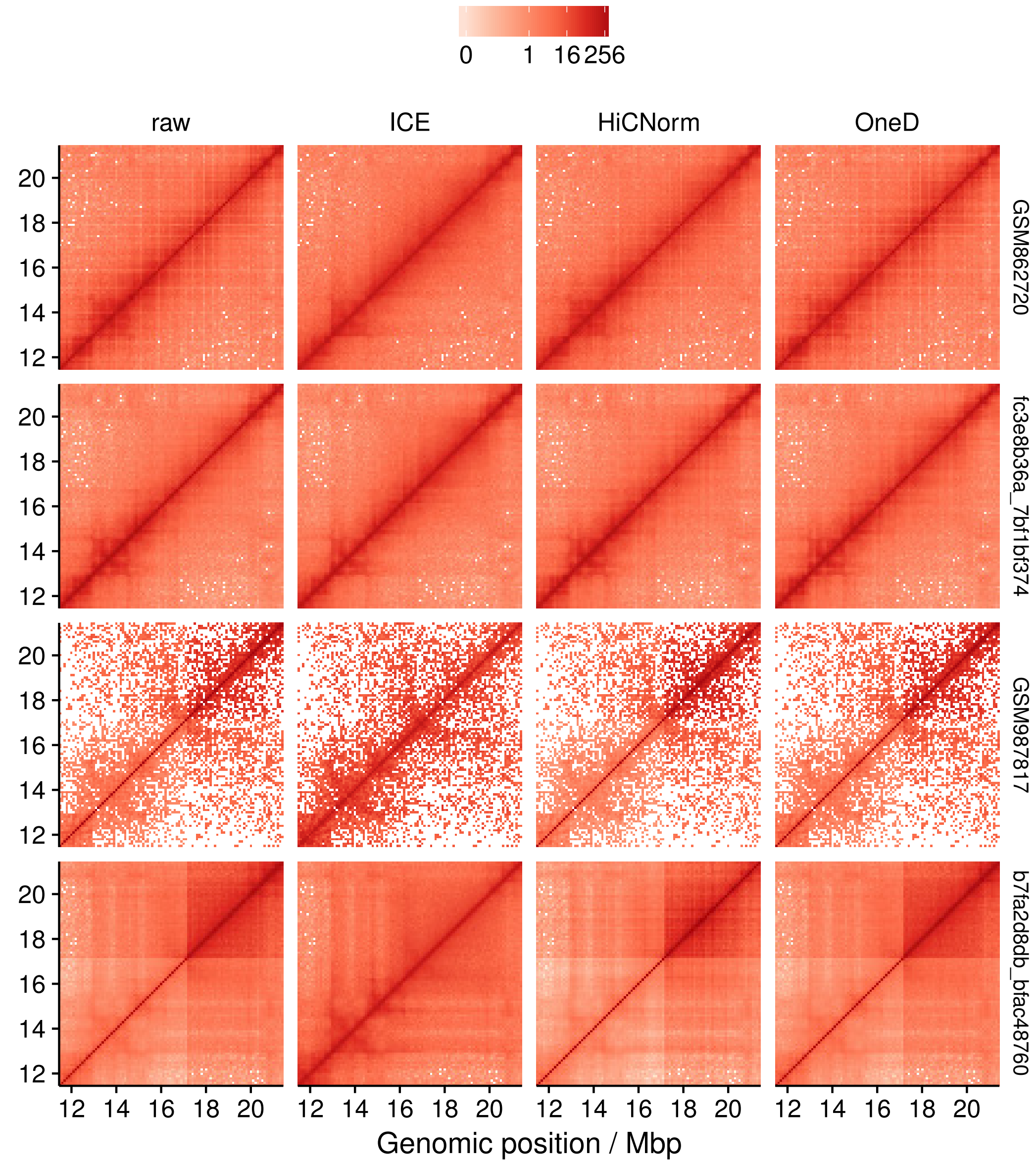

Figure S13: Detail of a representative example of the simulated Hi-C matrices (chr18). The contact matrices matrices at the upper two rows were simulated from ES cells and the lower two rows from B cells

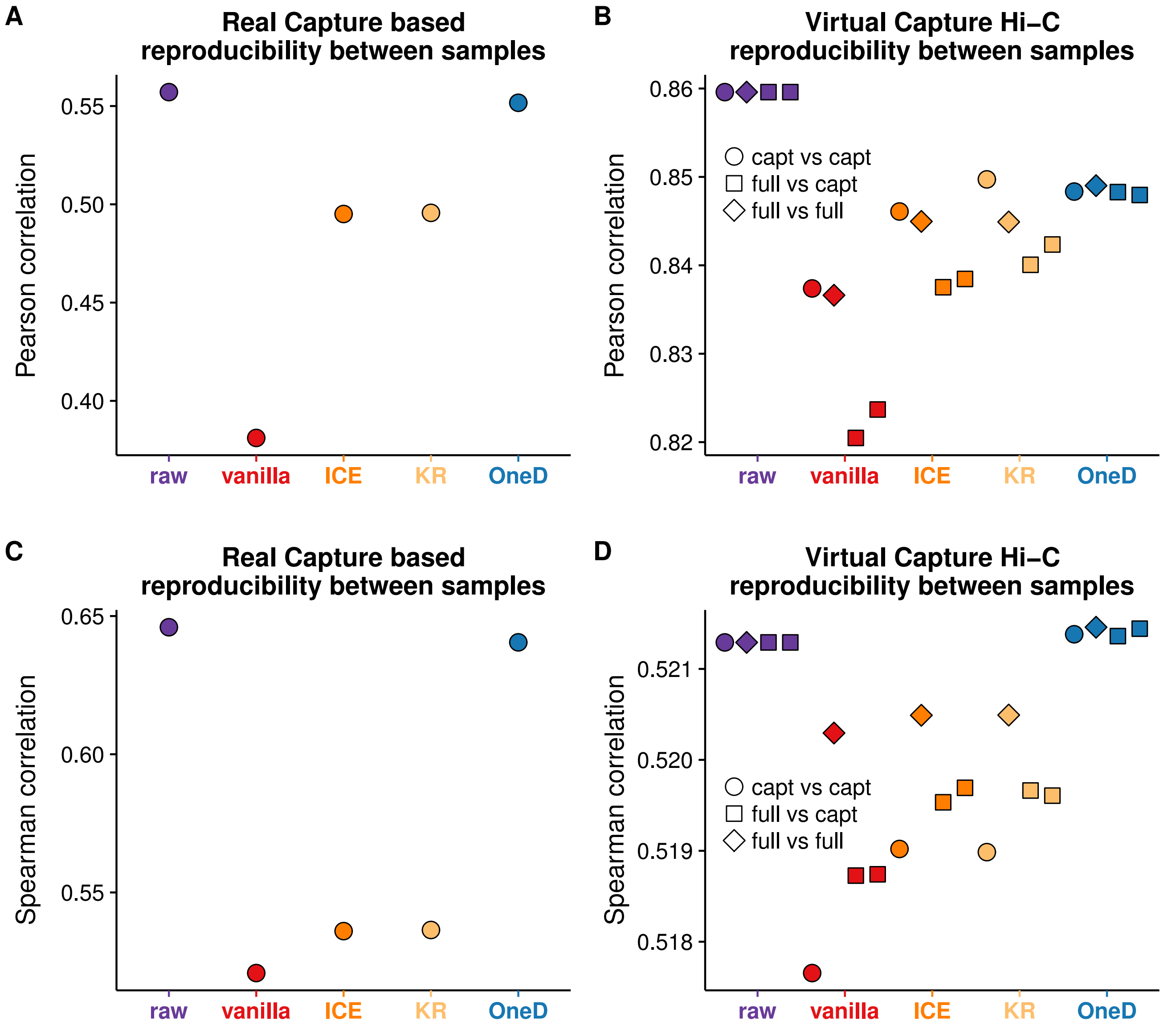

Figure S14: Bias removal on Capture Hi-C data A and C. Performance on the Capture Hi-C data. Two replicates centered on a 6 Mbp domain on chromosome 6 in T47D cells were normalized by the indicated tools and their Pearson (A) or Spearman (C) correlation computed. B and D. Performance on virtual Capture Hi-C data. Two Hi-C experiments were post-processed to produce an output similar to the Capture Hi-C experiment depicted in panels A and B. The signal outside the domain on chromosome 6 was removed and the data were normalized (capt), or the data were normalized before removing the signal outside the domain on chromosome 6 (full). Four matrices per method were generated and the Pearson (A) or Spearman (C) correlation computed between the pairs from different replicates were computed. Note that the scores are identical for the raw data because in this case the matrices of the same replicate are identical.

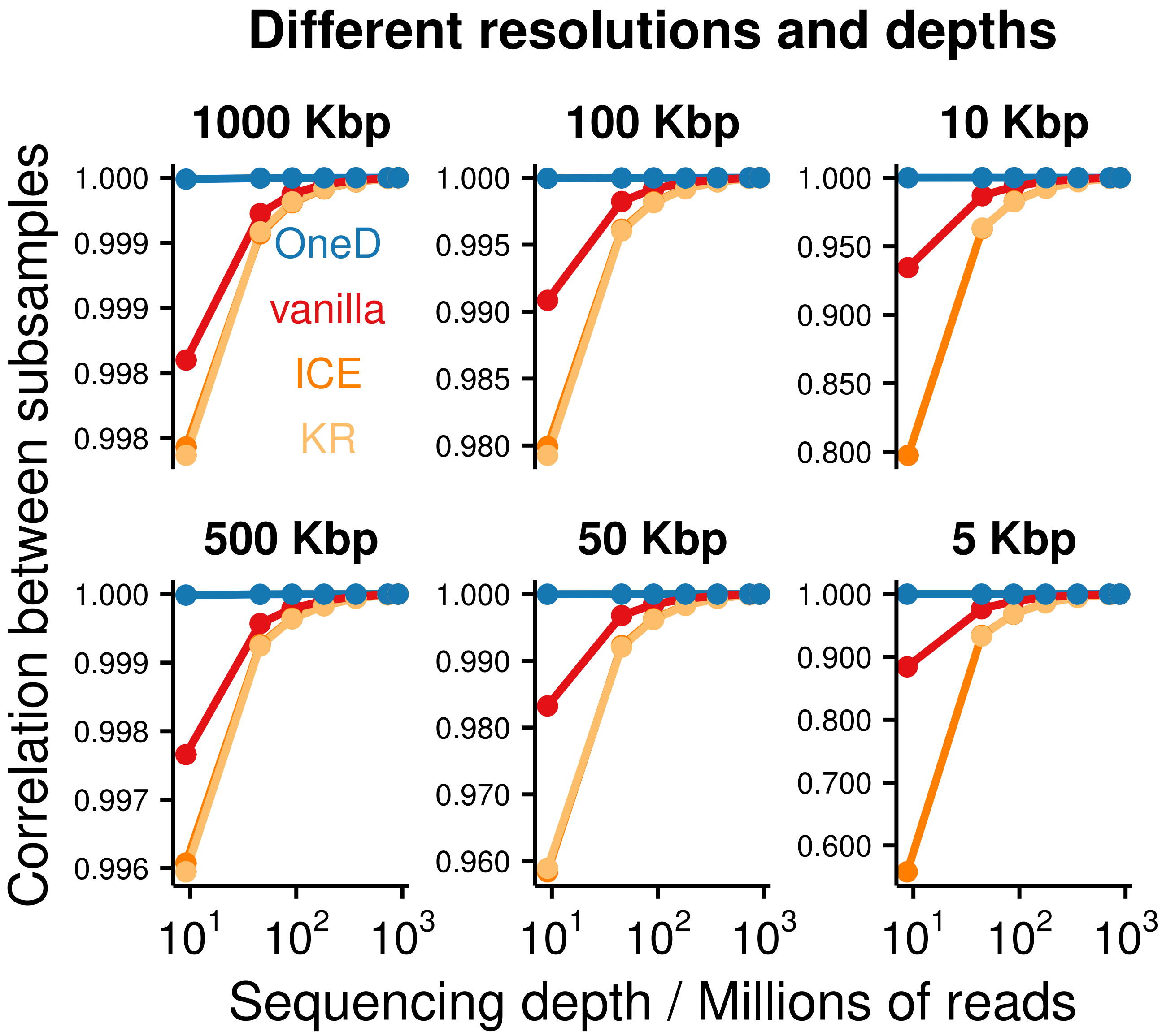

Figure S15: Correlation between subsamples at different resolutions and depths. X axis: sequencing depth. Y axis: Pearson correlation between the correction vectors of two different subsamples. Each color represents a correction method and each panel a bining resolution. Note the Y axis limits are specific to each panel.
